# Significance of serum branched-chain amino acid to tyrosine ratio measurement in athletes with high skeletal muscle mass

**DOI:** 10.1186/s13102-020-00229-1

**Published:** 2021-01-04

**Authors:** Katsuhiko Tsunekawa, Ryutaro Matsumoto, Kazumi Ushiki, Larasati Martha, Yoshifumi Shoho, Yoshimaro Yanagawa, Hirotaka Ishigaki, Akihiro Yoshida, Osamu Araki, Kiyomi Nakajima, Takao Kimura, Masami Murakami

**Affiliations:** 1 Department of Clinical Laboratory Medicine, Gunma University Graduate School of Medicine, Maebashi, 371-8511 Japan; 2 Graduate School of Health and Sport Science, Nippon Sport Science University, Yokohama, 227-0033 Japan; 3 Faculty of Education, Ikuei University, Takasaki, 370-0011 Japan; 4 Department of Medical Technology, Faculty of Health Science, Gunma Paz University, Takasaki, 370-0006 Japan

**Keywords:** Amino acid imbalance, Branched-chain amino acid to tyrosine ratio (BTR), Skeletal muscle index (SMI), Albumin, Thyroid hormone

## Abstract

**Background:**

Few nutritional markers reflect the hypermetabolic state of athletes with high levels of skeletal muscle. Although branched-chain amino acids (BCAA) play crucial roles in protein metabolism in skeletal muscle, the relationship between skeletal muscle mass and amino acid imbalances caused by the metabolism of BCAA and aromatic amino acids remains unclear. The aim of this study is to test the hypothesis that athletes with high levels of skeletal muscle mass have plasma amino acid imbalances, assessed by serum BCAA to tyrosine ratio (BTR) which can be measured conveniently.

**Methods:**

The study enrolled 111 young Japanese men: 70 wrestling athletes and 41 controls. None of them were under any medications, extreme dietary restrictions or intense exercise regimens. Each participant’s body composition, serum concentrations of albumin and rapid turnover proteins including transthyretin and transferrin, BTR, and thyroid function were assessed.

**Results:**

Compared to the controls, the athletes had significantly higher skeletal muscle index (SMI) (*p* < 0.001), and lower serum albumin concentration (*p* < 0.001) and BTR (*p* < 0.001). Kruskal–Wallis tests showed that serum albumin concentration and BTR were significantly lower in the participants with higher SMI. Serum albumin concentration and BTR were inversely correlated with SMI by multiple regression analysis (logarithmic albumin, β = − 0.358, *p* < 0.001; BTR, β = − 0.299, *p* = 0.001). SMI was inversely and transthyretin was positively correlated with serum albumin (SMI, β = − 0.554, *p* < 0.001; transthyretin, β = 0.379, *p* < 0.001). Serum concentration of free 3,5,3′-triiodothyronine (FT_3_) was inversely correlated with BTR, and, along with SMI and albumin, was independent predictor of BTR (SMI, β = − 0.321, *p* < 0.001; FT_3_, β = − 0.253, *p* = 0.001; logarithmic albumin, β = 0.261, *p* = 0.003). However, FT_3_ was not correlated with SMI or serum albumin. Serum concentrations of rapid turnover proteins were not correlated with BTR.

**Conclusions:**

Increased skeletal muscle mass enhances the circulating amino acid imbalances, and is independently facilitated by thyroid hormones. Serum BTR may be a useful biomarker to assess the hypermetabolic state of wrestling athletes with high levels of skeletal muscle.

## Background

Because of their branched structure, the essential amino acids valine, leucine, and isoleucine are collectively referred to as the branched-chain amino acids (BCAAs). These amino acids play crucial roles in skeletal muscle [1], not only as a major component of proteins, but also as an energy source, especially during exercise [1, 2]. BCAAs are also involved in the regulation of protein metabolism in skeletal muscle cells; for example, leucine activates mammalian target of rapamycin complex 1 (mTORC1), which stimulates protein synthesis and suppresses proteolysis by autophagy [3]. This activation of mTORC1 requires a high concentration of circulating leucine to be maintained [4].

Through training, sports athletes, who require instantaneous power, such as wrestlers, aim to increase their skeletal muscle mass for more effective energy use and improved competitive performance. Exercise induces an increase in whole-body energy expenditure and a prompt decrease in circulating BCAAs [5]. Several studies have reported that BCAA supplementation reduces muscle damage and protein breakdown during exercise through immediate increase in circulating BCAAs [6–8]. The changes in BCAA concentrations, whether decreased by exercise or increased by supplements, return to their initial levels through overnight [5]. However, the relationship between skeletal muscle mass and protein metabolism, including circulating BCAAs, has not been fully understood. In circulating lipid metabolism, lipoprotein lipase (LPL) plays a crucial role in triglyceride (TG)-rich lipoprotein hydrolysis [9]. LPL is highly expressed and synthesized in skeletal muscle tissues to use fatty acids for energy and translocated to the capillary lumen by glycosylphosphatidylinositol anchored high-density lipoprotein binding protein 1 (GPIHBP1), which has a pivotal role in the lipolytic processing of LPL [10]. We previously reported that wrestling athletes with high levels of skeletal muscle had high concentrations of LPL and GPIHBP1, and that increasing skeletal muscle mass improved effective energy use by promoting the hydrolysis of TG-rich lipoproteins [11].

Thyroid hormones also play a vital role in the energy metabolism in skeletal muscle [12]. They increase oxygen consumption and resting metabolic rate through increased mitochondrial activity related to the stimulation of mitochondrial enzymes and uncoupling protein 3 [13]. Thyroid hormones also promote skeletal muscle differentiation and induce the transition of slow fibers to fast fibers by the suppression of *Myh7* gene expression and through the stimulation of *Myh1*, *Myh2*, and *Myh4* expression [13]. However, the role of thyroid hormones in amino acid metabolism in skeletal muscle, especially that of BCAAs, has not been elucidated.

Metabolic turnover is based on a balance between the processes of synthesis and breakdown. Nutritional indicators, such as levels of albumin and rapid turnover proteins, are often used as blood biomarkers for assessing the condition of athletes. Although these markers reflect the ability of hepatic protein synthesis, few markers reflect the hypermetabolic state of athletes with high levels of skeletal muscle. Circulating amino acid imbalances, defined as elevated levels of aromatic amino acids (AAAs) such as tyrosine and phenylalanine, and/or decreased levels of BCAAs in plasma, are common in patients with liver failure [14]. These imbalances are caused by decreased metabolism of AAAs in the liver and increased metabolism of BCAAs in skeletal muscle with enhanced proteolysis causing sarcopenia [14, 15]. Therefore, the Fisher’s ratio, calculated by circulating BCAAs/AAAs, has been used as an indicator of the plasma amino acid imbalances in patients with liver diseases [16]. Concentrations of circulating AAAs and BCAAs are typically detected by amino acid analysis using liquid chromatography–mass spectrometry, which is cumbersome and not widely available [17]. In contrast, serum BCAAs to tyrosine ratio (BTR), which provides a simple indication of Fisher’s ratio, is often used in the clinical setting as a predictor for hepatocellular carcinoma in liver cirrhosis as well as an indicator of hepatic functional reserve [18], because serum BTR can be measured conveniently using a non-dedicated automated analyzer in a general hospital laboratory [19]. It is hypothesized that athletes with preserved liver function have circulating amino acid imbalances due to excessive levels of skeletal muscle. If the relationship between circulating amino acid imbalances, assessed by serum BTR, and skeletal muscle mass can be clarified in athletes, it may be possible to use the serum BTR as a biomarker of the hypermetabolic state. In addition, clarification of the relationship between the serum BTR and thyroid function tests could help elucidation of the novel mechanisms of BCAA metabolism via thyroid hormones in skeletal muscle.

The aim of this study was to test the hypothesis that athletes have plasma amino acid imbalances, assessed by serum BTR, due to excessive levels of skeletal muscle mass. We investigated the associations between serum BTR, skeletal muscle mass, and thyroid function in young Japanese men, including wrestling athletes with high skeletal muscle mass, and verified the usefulness of BTR measurement for these athletes.

## Methods

### Participants

This study was a subanalysis of a previous cross-sectional study [11]. In brief, we enrolled 111 young, healthy Japanese: 70 elite wrestling athletes and 41 college students who did not engage in habitual hard exercise. None were taking medications for metabolic diseases. All the participants provided written informed consent before being included in the study. The study was approved by the ethics committee of the Gunma University Graduate School of Medicine (approval no. 13–36).

### Physical examinations

The athletes were assessed at a time when they were not under any dietary restriction or undergoing intense training for a tournament. Blood samples were collected and physical examinations were performed in the morning after a 12-h fast period without exercise and BCAA supplementation. A bioimpedance instrument (InBody 430; InBody Japan, Tokyo, Japan) was used to measure body weight, fat mass, and skeletal muscle mass, with the participant in the standing position under the same conditions with minimal environmental effects including atmospheric pressure and humidity. The following indices were calculated: body mass index (BMI) as weight/height squared; fat mass index (FMI) as fat mass/height squared; skeletal muscle index (SMI) as skeletal muscle mass/height squared. Intra-assay coefficients of variation (CV) were 0% for BMI, 1.8% for FMI, and 0.5% for SMI, respectively.

### Thyroid function, serum concentrations of albumin and rapid turnover proteins, and BTR

With the participant in the sitting position, blood samples were collected from the antecubital vein using 23-G needles [11]. The serum samples were separated by centrifugation (1500×g) at 4 °C for 10 min and were stored at − 80 °C until analysis [11]. A LABOSPECT 008 automatic analyzer (Hitachi, Tokyo, Japan) was used to measure serum albumin concentrations using the modified bromocresol purple method, and serum transthyretin and transferrin concentrations using turbidimetric immunoassays. Intra- and inter-assay CV were 0.5–0.7 and 1.5% for albumin, 1.3–1.8% and 0.6–1.1% for transthyretin, and 0.8–1.2% and 1.3–1.4% for transferrin, respectively. A chemiluminescent microparticle immunoassay on an Abbott ARCHITECT i2000SR Immunoassay Analyzer (Abbott Laboratories, Abbott Park, IL, USA) was used to analyze serum concentrations of free 3,5,3′-triiodothyronine (FT_3_), free thyroxine (FT_4_), and thyrotropin (TSH). Intra- and inter-assay CV were 5.1–5.7% and 0.5–2.8% for FT_3_, 3.6–4.5 and 4.1% for FT_4_, and 2.3–4.4% and 2.8–4.1% for TSH, respectively. Serum BTR was measured by the enzymatic method using JCA-BM6050 automatic analyzer (JOEL, Tokyo, Japan) at LSI Medience Co. (Tokyo, Japan). Intra- and inter-assay CV of BTR were 0.2–0.3% and 0.7–1.0%, respectively.

### Statistical analysis

The data is expressed as median values with 25th–75th percentiles. Mann–Whitney *U* tests were used, as appropriate, to identify statistically significant differences between the two study groups. Kruskal–Wallis tests with Bonferroni multiple comparison tests were performed to compare the two groups classified by quartile. Spearman’s correlation analyses were performed to evaluate the relationships between SMI and the clinical variables, and between serum albumin concentrations or BTR and the clinical variables. Multiple regression analysis was performed to independently evaluate the indicators correlated to SMI, serum albumin concentration, or serum BTR. Shapiro-Wilk tests were used to evaluate the normal distribution. BMI, FMI, and serum albumin concentration were not normally distributed, thus, logarithmic transformation was performed for multiple regression analyses. Differences and correlations were considered significant when *p* < 0.05. SPSS Statistics version 25.0 (IBM Corp., Armonk, NY, USA) was used for the statistical analyses.

## Results

### Clinical characteristics of the participants

Table 1 presents the clinical characteristics of the control participants and wrestling athletes. Compared to the control group, the wrestling athlete group had significantly higher body weights (*p* = 0.012), BMI (*p* < 0.001), and SMI (*p* < 0.001), and lower FMI (*p* = 0.024), as reported previously [11]. The athlete group had significantly lower serum concentrations of albumin (*p* < 0.001), and BTR (*p* < 0.001). Serum concentrations of transferrin tended to be lower in the athlete group, although this difference was not statistically significant (*p* = 0.071). There were no differences in thyroid function tests between the groups.

**Table 1:** Comparison of clinical characteristics between the wrestling athletes and controls

	All participants		Control participants		Wrestling athletes		*p*
	(*N* = 111)		(*N* = 41)		(*N* = 70)		
Weight (kg)	66.6	(62.3–74.2)	64.2	(58.1–68.4)	68.4	(63.6–75.9)	0.012
BMI (kg/m^2^)	23.7	(22.2–25.6)	21.7	(20.1–24.0)	24.2	(23.2–26.0)	< 0.001
SMI (kg/m^2^)	11.9	(11.0–12.5)	10.5	(9.9–11.5)	12.4	(11.9–13.0)	< 0.001
FMI (kg/m^2^)	2.7	(2.3–3.7)	3.2	(2.3–4.9)	2.6	(2.2–3.5)	0.024
Albumin (g/dL)	4.5	(4.3–4.8)	4.8	(4.7–5.0)	4.3	(4.2–4.5)	< 0.001
Transthyretin (mg/dL)	29.8	(26.5–33.0)	29.8	(25.3–32.2)	29.8	(27.1–33.1)	0.790
Transferrin (mg/dL)	248	(228–268)	255	(235–272)	247	(222–264)	0.071
FT_3_ (pg/mL)	3.22	(3.09–3.39)	3.18	(3.04–3.34)	3.23	(3.10–3.41)	0.115
FT_4_ (ng/dL)	1.04	(0.98–1.10)	1.02	(0.98–1.10)	1.05	(0.97–1.11)	0.318
TSH (μIU/mL)	1.78	(1.24–2.25)	1.84	(1.19–2.12)	1.67	(1.36–2.30)	0.647
BTR	6.49	(5.80–7.58)	7.62	(6.83–8.61)	6.07	(5.51–6.64)	< 0.001

Data is expressed as the median (25th–75th percentile)

Mann-Whitney *U* test was used to compare the wrestling athletes with the control participants

*BMI* Body mass index; *SMI* Skeletal muscle index; *FMI* Fat mass index; *FT*_*3*_ Free 3,5,3′-triiodothyronine; *FT*_*4*_ Free thyroxine; *TSH* Thyrotropin; *BTR* Branched-chain amino acid to tyrosine ratio

### Skeletal muscle mass, serum albumin concentrations, and BTR

**Fig. 1:**
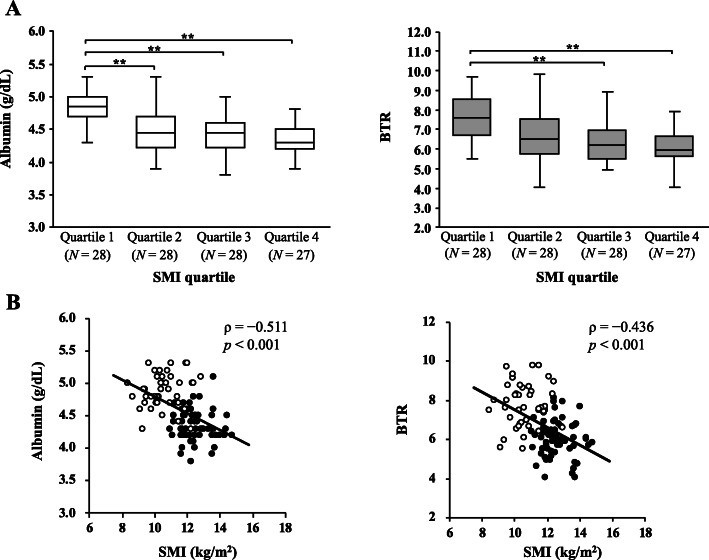
Association between skeletal muscle mass and serum albumin concentration or BTR (*N* = 111). Comparisons of serum albumin concentration and branched-chain amino acid to tyrosine ratio (BTR) between the quartiles for skeletal muscle index (SMI) (**a**). The SMI quartiles were as follows: Quartile 1, SMI ≤ 11.0 kg/m^2^; Quartile 2, 11.0 < SMI ≤ 11.9 kg/m^2^; Quartile 3, 11.9 < SMI ≤ 12.5 kg/m^2^; and Quartile 4, SMI > 12.5 kg/m^2^. The groups were compared with Kruskal–Wallis tests and Bonferroni multiple comparison tests (*p < 0.05, **p < 0.01). Results of the Spearman’s correlation analyses between SMI and serum albumin concentration or BTR (**b**). Open circles represent control participants and closed circles represent wrestling athletes

Figure 1 and Table 2 show the associations between SMI and serum albumin concentrations or BTR for all the participants. Figure 1a shows the comparisons of serum albumin concentration and BTR among the four groups classified by quartile for SMI. Quartiles 1 included only control participants, Quartiles 2 was composed of 68% athletes and 32% controls, Quartiles 3 was composed of 89% athletes and 11% controls, and Quartiles 4 was composed of 96% athletes and 4% controls. In each case, there were significant differences among the quartiles (serum albumin concentration, *p* < 0.001; BTR, *p* < 0.001; Kruskal–Wallis tests). In the Bonferroni multiple comparison tests, the serum albumin concentrations were significantly lower in Quartiles 2, 3, and 4 than in Quartile 1 for SMI. Similarly, BTR was significantly lower in Quartiles 3 and 4 than in Quartile 1 for SMI. The Spearman’s correlation analyses showed that serum albumin concentration and BTR was inversely correlated with SMI (albumin, ρ = − 0.511, *p* < 0.001; BTR, ρ = − 0.436, *p* < 0.001) (Fig. 1b). In contrast, SMI was not significantly correlated with serum concentrations of transthyretin (ρ = 0.121, *p* = 0.206), transferrin (ρ = − 0.157, *p* = 0.100), FT_3_ (ρ = 0.118, *p* = 0.216), FT_4_ (ρ = 0.043, *p* = 0.656), and TSH (ρ = 0.057, *p* = 0.552) (data not shown). Multiple regression analysis revealed that logarithmic albumin and BTR independently had significant correlations with SMI (logarithmic albumin, β = − 0.358, *p* < 0.001; BTR. β = − 0.299, *p* = 0.001) (Table 2).

**Table 2:** Independent predictors of SMI identified by multiple regression analysis (*N* = 111)

	SMI	
Variable	β	*p*
log Albumin (g/dL)	−0.358	< 0.001
BTR	−0.299	0.001

*SMI* Skeletal muscle index; *log Albumin* Logarithmic albumin; *BTR* Branched-chain amino acid to tyrosine ratio

### Correlations between serum albumin concentrations or BTR and clinical variables

**Fig. 2:**
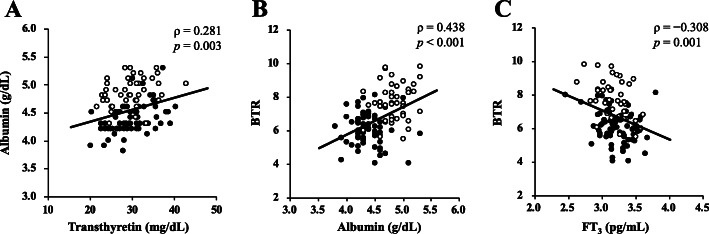
Correlations among serum albumin, BTR, and clinical variables (*N* = 111). Results of the Spearman’s correlation analyses between serum albumin concentration and transthyretin (**a**), between BTR and serum albumin concentration (**b**), and between BTR and FT_3_ (**c**). Open circles represent control participants and closed circles represent wrestling athletes

Tables 3 and 4 show the correlations between serum albumin concentration or BTR and the clinical variables. Spearman’s correlation analysis revealed that serum albumin concentration was positively correlated with transthyretin (ρ = 0.281, *p* = 0.003), transferrin (ρ = 0.299, *p* = 0.001), and BTR (ρ = 0.438, *p* < 0.001), and inversely correlated with body weight (ρ = − 0.213, *p* = 0.025) and BMI (ρ = − 0.370, *p* < 0.001), as well as with SMI (ρ = − 0.511, *p* < 0.001). There were no significant correlations between serum albumin concentration and the thyroid function tests. Multiple regression analysis revealed that SMI and transthyretin independently had significant correlations with logarithmic albumin (SMI, β = − 0.554, *p* < 0.001; transthyretin, β = 0.379, *p* < 0.001), but logarithmic BMI, transferrin and BTR did not. Figure 2a shows the positive correlation between the serum concentrations of transthyretin and albumin. Furthermore, BTR was positively correlated with FMI (ρ = 0.238, *p* = 0.012), and inversely correlated with body weight (ρ = − 0.214, *p* = 0.024), BMI (ρ = − 0.217, *p* = 0.022), SMI (ρ = − 0.436, *p* < 0.001), and FT_3_ (ρ = − 0.308, *p* = 0.001), but was not correlated with transthyretin and transferrin. Multiple regression analysis revealed that SMI, FT_3_, and logarithmic albumin had independent significant correlations with BTR (SMI, β = − 0.321, *p* < 0.001; FT_3_, β = − 0.253, *p* = 0.001; logarithmic albumin, β = 0.261, *p* = 0.003), but logarithmic BMI and FMI did not. Figure 2b shows the positive correlation between serum albumin concentration and BTR, while Fig. 2c illustrates the inverse correlation between serum FT_3_ concentration and BTR.

**Table 3:** Spearman’s correlation analyses between serum albumin concentration or BTR and clinical variables (*N* = 111)

	Albumin		BTR	
Variable	ρ	*p*	ρ	*p*
Weight (kg)	−0.213	0.025	−0.214	0.024
BMI (kg/m^2^)	−0.370	< 0.001	−0.217	0.022
SMI (kg/m^2^)	−0.511	< 0.001	−0.436	< 0.001
FMI (kg/m^2^)	0.104	0.279	0.238	0.012
Transthyretin (mg/dL)	0.281	0.003	0.172	0.072
Transferrin (mg/dL)	0.299	0.001	0.078	0.418
FT_3_ (pg/mL)	0.013	0.896	−0.308	0.001
FT_4_ (ng/dL)	0.107	0.262	−0.077	0.421
TSH (mIU/mL)	0.123	0.199	−0.019	0.846
BTR	0.438	< 0.001		

*BMI* Body mass index; *SMI* Skeletal muscle index; *FMI* Fat mass index; *FT*_*3*_ Free 3,5,3′-triiodothyronine; *FT*_*4*_ Free thyroxine; *TSH* Thyrotropin; *BTR* Branched-chain amino acid to tyrosine ratio

**Table 4:** Independent predictors of logarithmic albumin and BTR identified by multiple regression analysis (*N* = 111)

	log Albumin	
Variable	β	*p*
SMI (kg/m^2^)	−0.554	< 0.001
Transthyretin (mg/dL)	0.379	< 0.001
	**BTR**	
**Variable**	**β**	***p***
SMI (kg/m^2^)	−0.321	< 0.001
FT_3_ (pg/mL)	−0.253	0.001
log Albumin (g/dL)	0.261	0.003

*log Albumin* Logarithmic albumin; *SMI* Skeletal muscle index; *FT*_*3*_ Free 3,5,3′-triiodothyronine; *BTR* Branched-chain amino acid to tyrosine ratio

## Discussion

This study investigated associations between skeletal muscle mass and nutritional indicators, including serum concentrations of albumin, rapid turnover proteins, and BTR. The associations between these indicators and thyroid function tests also assessed as well. The wrestling athletes with higher levels of skeletal muscle mass had significantly lower serum albumin concentrations and BTR than the control participants. In all the participants, serum albumin concentration and BTR were inversely correlated with SMI, and serum FT_3_ concentration was inversely correlated with serum BTR, independently.

Previous reports have compared concentrations of circulating albumin and rapid turnover proteins between athletes and controls. Serum albumin concentrations tended to be lower in male professional cyclists and skiers than in controls [20]. Among athletes, rowers had lower plasma albumin concentrations than intermittent fasted athletes, such as Ramadan-fasted runners and boxers, and lightweight rowers had lower albumin concentrations than heavyweight rowers [21]. Serum transthyretin concentrations were higher in elite marathon runners than in controls, but there were no differences in retinol binding protein or transferrin concentrations between these groups [22]. Serum concentrations of albumin, transthyretin, and transferrin are used as the indicators of nutrition status and liver synthesis status in patients with various diseases, and generally have a positive correlations with this [23]. These indicators are often used to assess nutritional status in athletes in maintaining their condition. However, these are also influenced by many exercise-related factors, including dehydration and inflammation, thus no conclusions have been made regarding the use of these indicators for assessing the conditions of athletes undergoing continuous training. Moreover, the relationship between skeletal muscle mass and these indicators in athletes also remains unclear. In elderly populations, low serum albumin concentrations have been correlated with sarcopenia, which is defined as low levels of skeletal muscle mass [24]. In contrast, we observed an inverse correlation between skeletal muscle mass and serum albumin concentrations. This may be because all the participants had sufficient amounts of skeletal muscle, and that the excess skeletal muscle mass in wrestling athletes also utilized albumin even in the resting state.

The relationship between levels of circulating BCAAs and the skeletal muscle mass of athletes in a resting state has not been investigated previously, although the effects of BCAA supplementation to reduce muscle damage and breakdown by exercise have been extensively studied in athletes [6–8]. In the present study, the wrestling athletes had lower serum albumin concentration and BTR than the controls; these variables were inversely correlated with skeletal muscle mass. Serum BTR and albumin concentration are positively correlated in patients with chronic hepatitis [25], and similar results were obtained in the athletes of the present study. Although serum albumin concentration was positively correlated with the levels of rapid turnover proteins, there was no significant correlation between serum BTR and rapid turnover proteins. These results suggest that, even at rest, albumin and BCAAs may be used as sources of protein synthesis in skeletal muscles to a greater extent than other marker proteins. The serum albumin concentration also reflects liver synthesis ability, while serum BTR reflects the amino acid imbalances caused by hepatic AAA metabolisms and skeletal muscle BCAA metabolisms [18]. The decrease in serum BTR in this study is thought to be because the excessive amount of skeletal muscle promoting BCAA utilization rather than changes in hepatic AAA metabolism in wrestling athletes with preserved liver function. In contrast, another study found that patients with chronic heart failure had lower serum BCAA concentrations and Fisher’s ratios than controls, with positive correlations between the values of their SMI and their BCAA concentrations and Fisher’s ratios [26]. In patients with chronic liver diseases, lower BTR was associated with decreased skeletal muscle mass [27]. In these studies, the diseases may have caused increased BCAA catabolism or insufficient BCAA intake, resulting in the amino acid imbalances due to decreased concentrations of circulating BCAAs, which in turn may reduce skeletal muscle mass. Although the present study showed a decrease in serum BTR with increased skeletal muscle mass, it was not designed to assess the differences between enhanced BCAA consumption and reduced BCAA release in skeletal muscles. Further studies are needed to determine whether the concentration of circulating BCAAs reflects energy expenditure in skeletal muscle, through a detailed assessment of BCAA kinetics and measurement of other biomarkers of skeletal muscle mass and function.

Muscle cross-sectional area is reportedly lower in elderly subclinical hypothyroid patients than in age-matched euthyroid controls [28]. A recent study in elderly Chinese euthyroid subjects reported that serum FT_3_ concentrations were positively correlated with appendicular skeletal muscle mass, handgrip strength, and the results of a short physical performance battery [29]. In that study, the FT_3_ concentrations were especially low in subjects with sarcopenia. The present study did not observe a correlation between skeletal muscle mass and thyroid function tests although, unlike the previous studies, ours involved young participants with adequate skeletal muscle mass. However, we did observe an inverse correlation between serum FT_3_ concentration and serum BTR. A study of rats with hyperthyroidism showed that leucine supplementation improved their swimming performance [30]; this suggests that thyroid hormones increase BCAA metabolism in skeletal muscle. However, the mechanisms of thyroid hormone involvement in amino acid imbalances or BCAA metabolisms have not yet been studied. Further studies are needed to elucidate the roles of thyroid hormones on BCAA metabolism in skeletal muscle.

This study had several limitations. Firstly, it had a relatively small sample size. We specifically enrolled elite wrestling athletes with high skeletal muscle mass at a time when they were not undergoing intense training or extreme dietary/water restrictions for a tournament. Secondly, this cross-sectional study evaluated the correlations between several different factors, but other exercise-related factors including hydration status and inflammation may also be involved. Thirdly, the participants’ diets and exercise prior to the sample collection were not fully standardized. It has been reported that plasma BCAA concentrations in healthy male students increase immediately after BCAA ingestion, peak at 30 min, and gradually decrease to the initial level by 180 min after ingestion [31]. Another study found that circulating BCAA concentrations decreased by a squat exercise session which consisted of 7 sets of 20 squats without BCAA supplementation return to their initial levels through overnight [5]. In the present study, nutritional status, including BCAA metabolism, may have been at baseline without any immediate influence in the athletes and the controls who did not exercise, eat meals or BCAA supplements for 12 h before sample collection. However, another study found that resistance physical exercise prolongs muscle protein synthesis stimulation approximately 24 h post-workout [32]. An assessment of the relationship between amino acid imbalances and skeletal muscle mass in athletes under a longer period than 24 h without exercise before sample collections will prove the validity of the present results. Lastly, further studies are needed to confirm our hypothesis through investigations of different athletes with various types of specialized skeletal muscle function, such as instantaneous power or endurance strength.

## Conclusions

This study showed that the serum albumin concentrations and BTR of young Japanese men were significantly lower in wrestling athletes with high levels of skeletal muscle than in controls. These variables were found to be inversely correlated with skeletal muscle mass. These results suggest that an increase in skeletal muscle mass enhances the imbalances in circulating amino acids, probably due to increased BCAA metabolisms in the skeletal muscle. The amino acid imbalances may also be increased by thyroid hormones independently. It may be possible to use BTR as a biomarker of hypermetabolic state in athletes with high levels of skeletal muscle mass.

## Data Availability

The datasets used and/or analyzed during the current study are available from the corresponding author on reasonable request.

## Abbreviations

BCAA: Branched-chain amino acid
BMI: Body mass index
BTR: Branched-chain amino acid to tyrosine ratio
FMI: Fat mass index
FT_3_: Free 3,5,3′-triiodothyronine
FT_4_: Free thyroxine
GPIHBP1: Glycosylphosphatidylinositol anchored high-density lipoprotein binding protein 1
LPL: Lipoprotein lipase
mTORC1: Mammalian target of rapamycin complex 1
SMI: Skeletal muscle index
TG: Triglyceride
TSH: Thyrotropin
